# Why Do Countries Regulate Environmental Health Risks Differently? A Theoretical Perspective

**DOI:** 10.1111/risa.13165

**Published:** 2018-08-15

**Authors:** Sander C. S. Clahsen, Irene van Kamp, Betty C. Hakkert, Theo G. Vermeire, Aldert H. Piersma, Erik Lebret

**Affiliations:** ^1^ Centre for Sustainability, Environment and Health National Institute for Public Health and the Environment—RIVM Bilthoven The Netherlands; ^2^ Institute for Risk Assessment Sciences Utrecht University Utrecht The Netherlands; ^3^ Centre for Safety of Substances and Products National Institute for Public Health and the Environment—RIVM Bilthoven The Netherlands; ^4^ Centre for Health Protection National Institute for Public Health and the Environment—RIVM Bilthoven The Netherlands; ^5^ National Institute for Public Health and the Environment—RIVM Bilthoven The Netherlands

**Keywords:** International differences in environmental health risk management, review of conceptual frameworks, science–policy interface

## Abstract

Why do countries regulate, or prefer to regulate, environmental health risks such as radiofrequency electromagnetic fields and endocrine disruptors differently? A wide variety of theories, models, and frameworks can be used to help answer this question, though the resulting answer will strongly depend on the theoretical perspective that is applied. In this theoretical review, we will explore eight conceptual frameworks, from different areas of science, which will offer eight different potential explanations as to why international differences occur in environmental health risk management. We are particularly interested in frameworks that could shed light on the role of scientific expertise within risk management processes. The frameworks included in this review are the Risk Assessment Paradigm, research into the roles of experts as policy advisors, the Psychometric Paradigm, the Cultural Theory of Risk, participatory approaches to risk assessment and risk management, the Advocacy Coalition Framework, the Social Amplification of Risk Framework, and Hofstede's Model of National Cultures. We drew from our knowledge and experiences regarding a diverse set of academic disciplines to pragmatically assemble a multidisciplinary set of frameworks. From the ideas and concepts offered by the eight frameworks, we derive pertinent questions to be used in further empirical work and we present an overarching framework to depict the various links that could be drawn between the frameworks.

## 1. INTRODUCTION

International differences in the management of environmental health risks occur frequently. For example, the United States and the European Union regulate potential risks posed by pesticide contaminants in drinking water differently. In the United States, a maximum contaminant level (MCL) is derived for a single pesticide, based on the available toxicological evidence and an analysis of the costs and health benefits associated to the proposed MCL (EPA, 2016). Alternatively, in the European Union, almost all pesticides approved for use are subject to the same maximally allowable concentration (MAC) of 0.1 μg/L, which is supposed to function as a precautionary‐based “surrogate zero” level (Dolan, Howsam, Parsons, & Whelan, 2013). Similar differences can be observed between European countries. Redmayne (2016) has shown that regulatory approaches toward children's exposure to radiofrequency EMFs differ widely among European countries. In addition, Löfstedt (2011) argues that several European countries have different preferences with regard to the management of the industrial chemicals Bisphenol A and Deca BDE.

In this theoretical review, we will explore various theoretical perspectives that help understand these international differences in risk management. We are interested in studying the reasoning behind countries' risk management strategies, and to better understand why these strategies differ between countries. To further specify our understanding of the term “international differences in risk management strategies,” we distinguish two dimensions in which risk management can differ across countries. First, countries can select different risks for regulation. As Wiener, Rogers, Hammitt, and Sand (2011) point out, there are key differences between the United States and Europe in terms of the policy areas that are thought to require precautionary regulatory interventions. Second, in the event that countries set out to manage the same risk issue, then differences in the stringency of the ultimate measures can occur. For example, Rothstein et al. (2017) found that there are variations in the stringency of occupational health and safety regulations in Germany, France, the United Kingdom, and the Netherlands. The theoretical frameworks discussed throughout this article could be used, although to different extents, to reflect on these two dimensions.

We are specifically interested in the role of scientific expertise within risk management processes. This interest comes from our working experience as experts in institutes and expert committees with a mission to translate scientific knowledge for environmental and public health policymakers. In this capacity, we have experienced that the relationship between science and policy is not as straightforward as the term “evidence‐based policy” seems to suggest. Decades of research into this relationship in the domain of environmental health risks theoretically and empirically underlines these experiences (Funtowicz & Ravetz, 1993; Hoppe, 2009; Jasanoff, 1990; Pielke, 2007; Sarewitz, 2004; Spruijt et al., 2014). The variety of conceptual frameworks discussed in this article shed light on various aspects of complex science–policy interactions.

As a result, a variety of explanations for international differences in risk management strategies are explored: Do the differences stem from different overall interpretations by experts of the underlying scientific evidence, that is, interpretative ambiguity (after Renn, 2008)? Do these differences occur because experts hold different views about the acceptability of risks, that is, normative ambiguity? Or does expert judgment only play a minor role in the final judgment and do differences emerge through influences from other stakeholders? Or do the differences follow from differences in (national) cultures? The outcomes of this review will be used to structure stakeholder interviews and document analyses used in further case studies that aim to study international differences in environmental health risk management.

Overall, the aim of the present review is to provide a theoretical foundation for empirical research that seeks to understand why countries frequently manage environmental health risks differently, and how expert policy advisors specifically, and scientific knowledge in general, are involved in the development of these different risk management strategies. To that end, we will discuss eight different conceptual frameworks: the Risk Assessment Paradigm, research into the advisory roles of experts in risk management processes, the Psychometric Paradigm, the Cultural Theory of Risk, several participatory approaches to risk assessment and risk management, the Advocacy Coalition Framework, the Social Amplification of Risk Framework, and Hofstede's Model of National Cultures.

## 2. METHODS

The selection of the eight conceptual frameworks included in this article occurred through a pragmatic rather than a systematic approach. A systematic selection of relevant frameworks would prove challenging because concepts and terminology vary across scientific domains. Through cognitive distance between experts from different domains, different words are used for (nearly) similar concepts and the same words for different concepts (Lebret, 2016). Moreover, in some academic fields, main thoughts and concepts are published in books rather than in peer‐reviewed journal articles or proceedings. Alternatively, we drew from various sources to identify relevant conceptual frameworks. First, a variety of theories and models has been brought forward in scientific debates occurring in literature, in conference discussions, in expert committee meetings and discussions occurring between relevant colleagues. Second, we were familiar with several conceptual frameworks through our knowledge and experiences in the fields of environmental health, food safety, regulatory toxicology, risk perception, risk governance, science and technology studies, and (organizational) psychology. Third, we used a “snowball” approach to identify further relevant frameworks.

## 3. RESULTS

### 3.1. Understanding the Concept of “Risk”

Because the concept of “risk” is referred to extensively throughout this article, we think clarity about our specific understanding of this concept is required. Indeed, “risk” can be defined in numerous ways (Aven & Renn, 2010). Accordingly, Rayner (1992) argues that “risk” is a “polythetic” concept, meaning that a wide variety of equally legitimate, though complementary and sometimes contrasting, understandings of the concept of “risk” coexist. In the present article, two influential though contrasting understandings of “risk” play a key role.

First, in the risk assessment sciences (i.e., toxicology, epidemiology, and exposure sciences), risks are interpreted in a quantitative and technical sense. Risks are typically assessed in terms of their physical characteristics, in a “probability of occurrence * magnitude of effects” fashion. The Risk Assessment Paradigm is the key intellectual foundation of this type of risk assessment.

Alternatively, risk perception research (i.e., research focusing on how others than subject‐matter experts typically assess risks) has revealed that other considerations than the physical characteristics of a risk often play an important role in risk evaluation. Two theoretical perspectives are dominant here: the Psychometric Paradigm and the Cultural Theory of Risk. Both perspectives refer to the “construction of risk” (although in different ways), meaning that risks are not considered fixed, objective entities interpreted the same by everyone. Rather, individuals and groups construct risks differently, depending on their personal values, social and cultural contexts and other economic, legal, and ethical considerations. On these grounds, one could also assert that technical risk assessment is a specific type of risk perception, developed, systematized, and maintained by expert risk assessors, in an effort to thoroughly characterize a risk in the most objective way possible.

### 3.2. Overview of Eight Conceptual Frameworks

#### 3.2.1. Risk Assessment Paradigm

**Figure 1:**
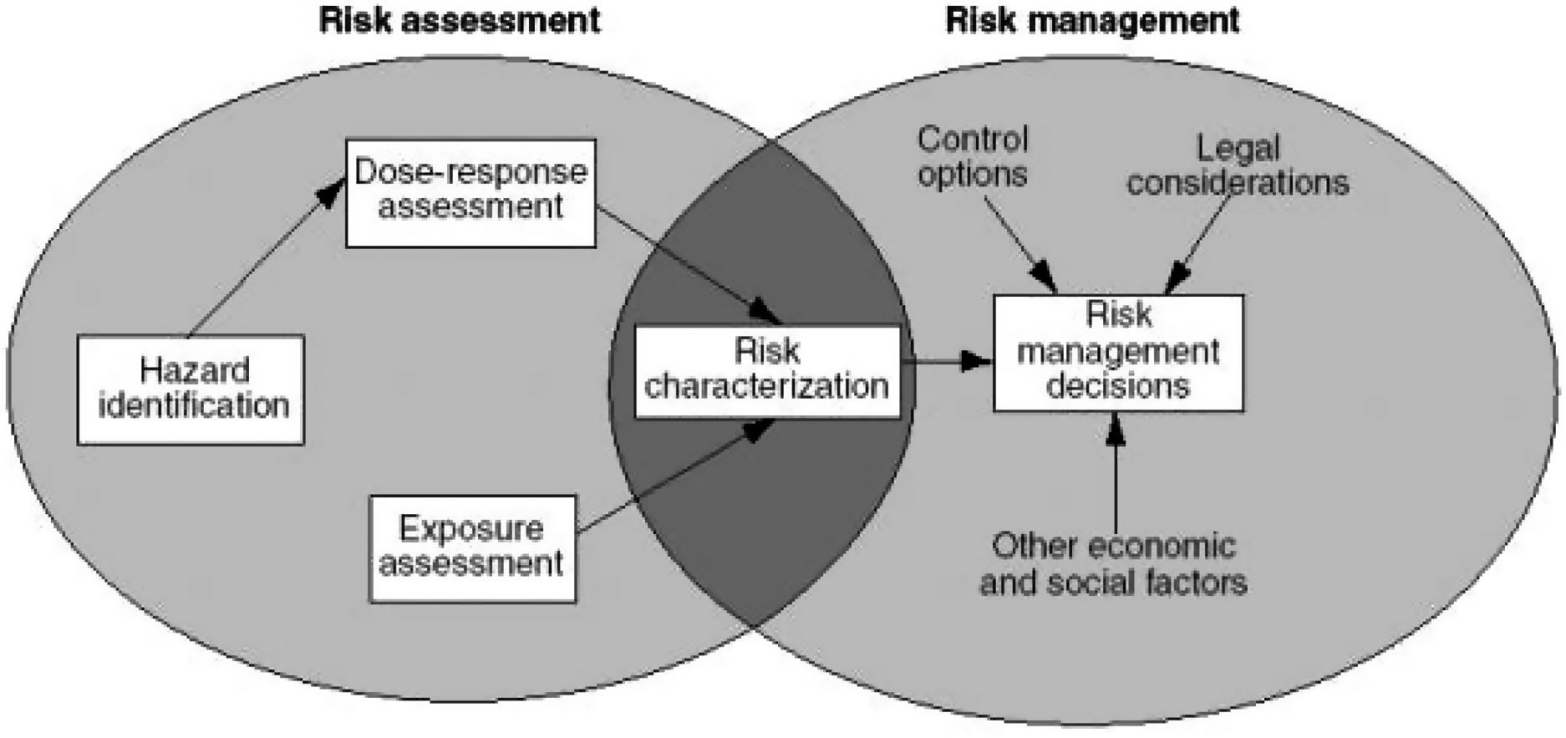
The Risk Assessment Paradigm as described in the NRC's 1983 “Risk Assessment in the Federal Government: Managing the Process.” *Source*: EPA, n.d. Permission to reprint this figure has been obtained from the copyright holder (U.S. Environmental Protection Agency).

Policymakers often require the assessment of a particular environmental health risk, such as risks associated with exposure to particulate matter, electromagnetic fields, or specific chemical substances. Such risk assessments are often performed in accordance with the Risk Assessment Paradigm (RAP), as originally outlined in the Red Book of the U.S.‐based National Research Council (NRC, 1983). In the IPCS Risk Assessment Terminology report (IPCS, 2004), “risk assessment” is defined as “a process intended to calculate or estimate the risk to a given target organism, system or (sub)population, including the identification of attendant uncertainties, following exposure to a particular agent, taking into account the inherent characteristics of the agent of concern as well as the characteristics of the specific target system” (p. 14). Four steps together form the risk assessment process: hazard identification, dose–response assessment, exposure assessment, and risk characterization; see also Fig. 1. According to a later report of the NRC, the main achievement of the 1983 version of the paradigm is its popularizing of the distinction of the risk assessment process from the process of risk management (NRC, 1996). Other, later models that describe or prescribe the process of risk assessment, such as the model developed by Covello and Merkhofer (1993), similarly maintain this distinction, signaling the influence of this idea. Nevertheless, in its later works (see NRC, 1996, 2009), the NRC recognized the need for risk assessors, risk managers, and other stakeholders to cooperate at various stages of the risk management process (e.g., the problem formulation stage), blurring the lines between risk assessment and risk management (see also Section 3.2.5).

From the point of view of this paradigm, regulatory differences can occur due to differing interpretations of scientific evidence by experts and different (methodological) choices made by experts in situations of uncertainty. In the environmental health domain, scientific knowledge pointing to a “high‐risk” situation can often coexist with knowledge suggesting a “low‐risk” situation. For example, experts disagree about the carcinogenic properties of glyphosate and, accordingly, about the risks associated with exposure to this herbicide (compare FAO/WHO, 2016 and EFSA, 2015 with IARC, 2017). In addition, Beronius, Rudén, Hakansson, and Hanberg (2010) note that the outcomes of the various risk assessments of Bisphenol A differ considerably, illustrating the apparent ambiguity over whether or not BPA is shown to pose a risk to (parts of) the population. The phenomenon that experts interpret certain scientific evidence differently is also known as *interpretative ambiguity* (Renn, 2008). As a result of this ambiguity, the degree to which either line of evidence is represented within a country may differ internationally; experts in country A may be pointing more to evidence suggesting a “high‐risk” situation, whereas experts in country B mostly refer to evidence suggesting a “low‐risk” situation when providing policy advice. In short, when studying international differences in risk management strategies, one could investigate which specific lines of (risk assessment) evidence were used to support the risk management process, and how the evidence referred to within these processes differs from country to country.

#### 3.2.2. Expert Advisory Roles in Risk Management Processes

When highly specialized, in‐depth knowledge is required to thoroughly understand the hazardousness of a risk, policymakers can turn to experts for science‐based policy advice. Traditionally, policymakers and experts should interact while observing a clear division of labor: policymakers are responsible for the normative process of policy development, whereas experts are responsible for the independent, value‐free process of scientific knowledge generation. The (conceptual) separation between risk assessment and risk management exemplifies this idea. However, this traditional separation of roles does not accord with the complex relationship between experts and policymakers as observed in reality (see e.g., Jasanoff, 1990). The roles of experts in policy processes may particularly become ambiguous when risks are complex, uncertain, and contested, like the risks associated with exposure to RF EMFs (Spruijt et al., 2014).

**Figure 2:**
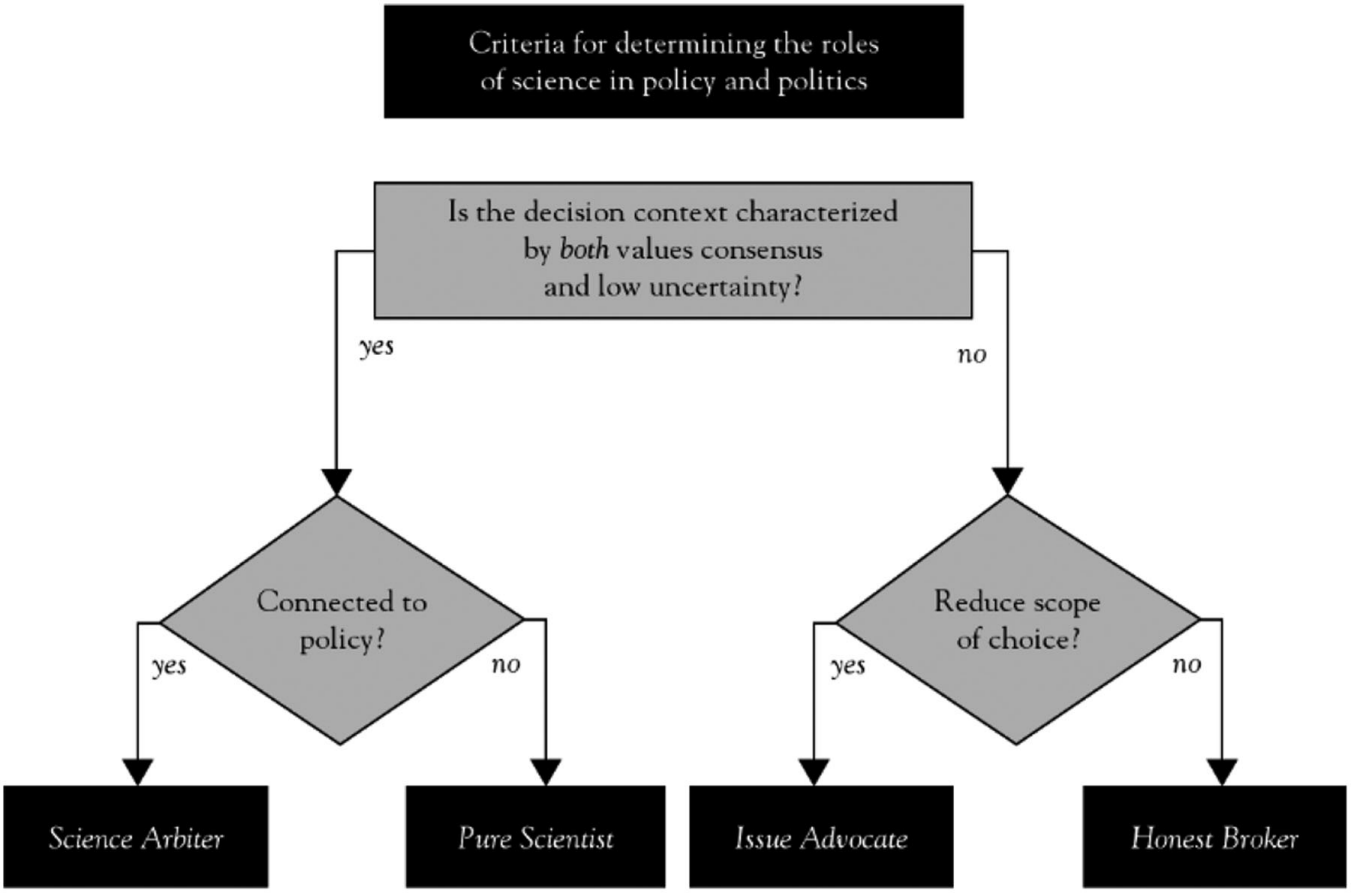
Four ideal typical roles developed by Pielke: Science Arbiter, Pure Scientist, Issue Advocate, and Honest Broker. *Source*: Pielke, 2007. Permission to reprint this figure is automatically granted by the copyright holder (Cambridge University Press) under condition of acknowledgment

We have identified several theoretical typologies of expert roles that describe the particular options (theoretically) available to experts when acting as a policy advisor. Pielke (2007) distinguishes four ideal‐typical expert roles (see also Fig. 2). These expert roles pertain to the degree of participation within policy processes (ranging from none at all to active participation) and the breadth of an expert's policy advice (advocacy of a particular measure or providing a range of policy alternatives). The *Pure Scientist* typically performs research out of intrinsic interest and does not interact with policymakers in any way whatsoever, whereas the *Science Arbiter* answers factual questions posed by policymakers. The *Issue Advocate* advocates the implementation of one specific policy option, thus reducing the scope of possible policy options, whereas the *Honest Broker* aims to provide an overview of all scientifically legitimate policy alternatives, thus broadening the scope of possible policy options. Pielke (2007) proposes that all four roles are equally legitimate, but the appropriateness of a specific role depends on the decision context, characterized by the uncertainty and values consensus surrounding the risk.

**Figure 3:**
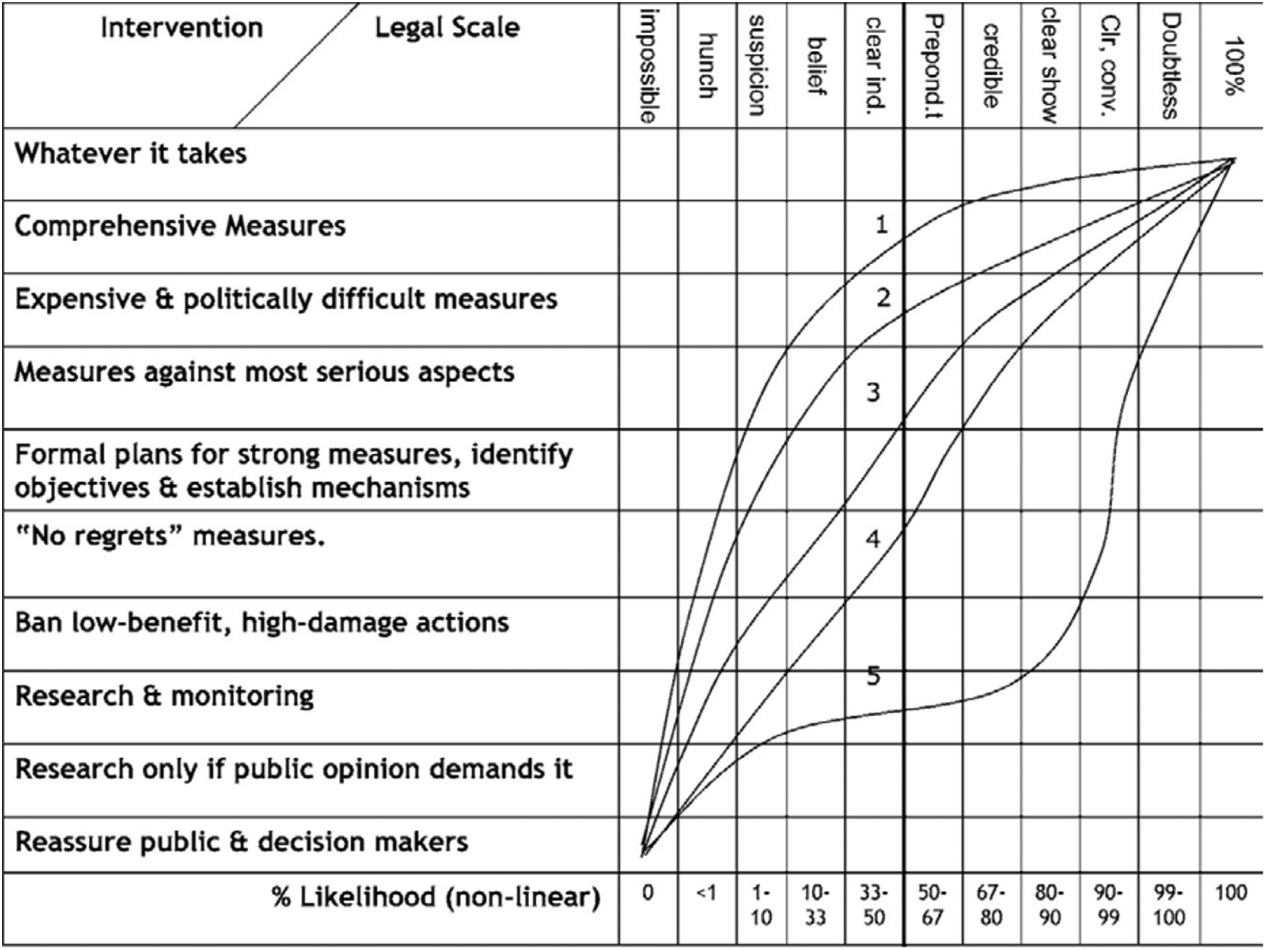
The level of intervention to address severe hazards to the environment is plotted against the level of scientific (un)certainty and five roles of experts. The probability scale is asymmetrical and nonlinear. The curves correspond to the following five expert roles: 1. Environmental Absolutist, 2. Cautious Environmentalist, 3. Environmental Centrist, 4. Technological Optimist, 5. Scientific Absolutist. *Source*: Weiss, 2003. Permission to reprint this figure has been obtained from the copyright holder (Springer Nature).

Furthermore, Weiss (2003) distinguishes five ideal‐typical expert roles (see also Fig. 3). These expert roles pertain to the technical contents of policy advice, particularly in relation to the level of (precautionary) action advocated, given a certain degree of scientific (un)certainty. The *Scientific Absolutist*, typically advocating “science‐based regulation,” requires high levels of scientific certainty before supporting “measures against the most serious aspects” of a technological development that may pose potential dangers to the environment. Alternatively, the *Environmental Absolutist*, typically advocating relatively early precautionary action, requires much less scientific certainty before advising the same measure, based on the norm that man should inherently protect the environment from potential (man‐made) dangers. The *Technological Optimist*, *Environmental Centrist*, and *Cautious Environmentalist* hold intermediate positions.

In an interdisciplinary review of the available literature on the roles of experts as policy advisors on complex issues, Spruijt et al. (2014) identified six factors that influence expert roles. These are: the type of issue, the type of knowledge of an expert, the core values of an expert, the organization in which the expert works, the wider context of the expert, and the changing beliefs of experts. Though this review included a wide variety of theoretical ideas from various scientific disciplines, Spruijt, Knol, Torenvlied, and Lebret (2013) acknowledge that empirical evidence supporting this theory is scarce. To fill this gap, Spruijt and colleagues studied the advisory roles of experts in the fields of EMF (Spruijt, Knol, Petersen, & Lebret, 2015), particulate matter (PM) (Spruijt, Knol, Petersen, & Lebret, 2016), and antimicrobial resistance (Spruijt, 2016). They found that advisory roles can vary strongly among experts of the same subject matter, though the identified expert roles did not precisely fit the Pielke and Weiss typologies. In short, participating EMF and PM experts had different judgments as to the necessity of additional (precautionary) measures in EMF risk management (Spruijt et al., 2015) and PM risk management (Spruijt et al., 2016). Furthermore, PM experts disagreed about whether it is their responsibility to act as an “issue advocate,” that is, recommend the policy option that the expert deems most suitable (Spruijt et al., 2016).

From the theoretical and empirical work on the roles of experts as policy advisors, we draw the main message that science–policy interactions are highly intricate by nature. A recurring theme is the role of normative values held by experts and how such values could (or should) influence their advisory roles. For example, the five expert roles developed by Weiss implicitly relate to normative positions about the importance of scientific knowledge in policy making, the vulnerability of environmental or human physiological systems, and the legitimacy of the Precautionary Principle as a guiding legislative principle. In turn, these expert roles draw attention to the possible variations in stringency of (precautionary) measures advised by expert policy advisors. When experts hold different value‐based positions with regard to the acceptability of a risk, this can be referred to as normative ambiguity (in contrast to interpretative ambiguity) (Renn, 2008). A key consequence of normative ambiguity in science is that it matters considerably which expert provides advice to policymakers. The idea that different experts may provide different policy advice could be used as an explanation for observed international differences in risk management.

#### 3.2.3. Psychometric Paradigm

**Figure 4:**
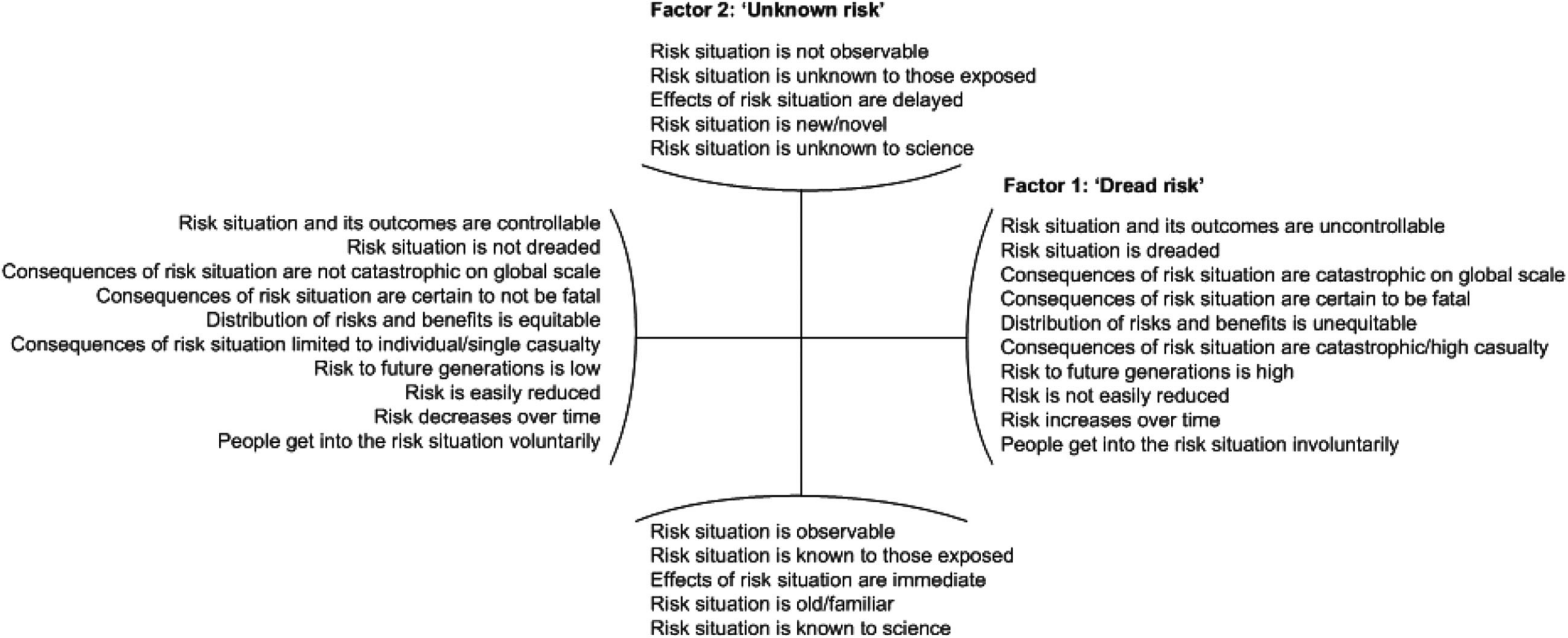
Overview of characteristics of risk that make up two key factors in the Psychometric Paradigm: dread risk and unknown risk. The third factor, number of people exposed, is not shown here.

In essence, research within the Psychometric Paradigm (PP) aims to unveil factors that determine the risk perception of people (Siegrist, Keller, & Kiers, 2005). This type of research is quantitative by nature, employing questionnaires to ask respondents about the perceived riskiness, acceptability, and the desired level of risk regulation for a wide variety of human activities and technologies (Slovic, 1987). Notably, these findings are then compared to the respondents' scorings on qualitative characteristics of risk that are hypothesized to influence risk perceptions. For example, Fischhoff, Slovic, Lichtenstein, Read, and Combs (1978) asked respondents to rate each of the 30 activities or technologies included in the study on nine characteristics of risk. These were: voluntariness of risk, immediacy of effect, knowledge about risk (exposed), knowledge about risk (science), control over risk, newness, chronic–catastrophic, common–dread, and severity of consequences. Research into the statistical relationships between these different characteristics of risk, by means of factor analysis, showed that some characteristics correlated highly with one another. In fact, three higher‐order factors have been discerned repeatedly: “dread risk,” “unknown risk,” and “number of people exposed” (Slovic, 1987; Slovic, Fischhoff, & Lichtenstein, 1976). The specific characteristics of risk that define these higher‐order factors can be observed in Fig. 4. Individual risks can then be placed in a two‐dimensional factor plot, in accordance with their scoring on the aggregated “dread risk” and “unknown risk” factors. The resulting factor plots, sometimes referred to as “cognitive maps,” represent the average risk perceptions of the entire group of respondents (Slovic, 1987).

The PP resulted from a research program aimed at studying cognitive processes that determined man's responses to risks, particularly natural hazards (Slovic, 1987). Attempts to explain why the perceptions of some hazards seemed to defy the outcomes of statistics‐based risk assessment (e.g., exemplified by societal resistance to statistically speaking low‐risk nuclear power) focused on the limitations of human cognition. According to Slovic et al. (1976), the idea that any decisionmaker is “boundedly rational” (Simon, 1957) functioned as a key source of inspiration here. In addition, Tversky and Kahneman (1974) showed, through various psychological laboratory studies, that humans often revert to heuristics, or mental shortcuts, to make judgments under uncertainty. For example, the availability heuristic could explain why the perceived probability of an event occurring increases when immediate and powerful cues (such as vivid images) are available (Tversky & Kahneman, 1973).

When applying insights from the PP to the study of regulatory differences between countries, the question immediately arises whether there were significant differences in societal risk perceptions, and whether there were major differences in the salience of the risk issue among the populations of the different countries. One would expect that, in a democratic society, public policymakers are reasonably responsive to the concerns of those confronted with the risky activity when developing a risk management strategy (see e.g., Slovic, Fischhoff, & Lichtenstein, 1982). Differences in countries' general societal risk perceptions could then be an explanation for international differences in risk management.

#### 3.2.4. Cultural Theory of Risk

The Cultural Theory of Risk (CTR) sets out to explain how individuals and groups select and interpret dangers (Tansey & O'Riordan, 1999). For example, Douglas and Wildavsky (1983) describe various reasons for why some people are specifically attentive to environmental risks, whereas others focus particularly on risks posed by violent crime. According to these authors, such risk selections (and subsequent risk perceptions) are shaped by the social context, and particularly by the *social organization* of the group in which the individual belongs. This premise is mainly supported by insights from anthropological research into the function of rules, symbolisms, and rituals in various “primitive” societies. Douglas (1966) describes a wide range of “pollution beliefs,” held by the society's members, about dangers that may be inflicted on a transgressor, his kin, or others by exhibiting certain purity‐ or sacredness‐defiling behavior. An example is the belief that a women's adultery causes bodily pains in the transgressor's husband. Because this particular society's layered structure is regulated by considerations like marriage payments and marital status, and adultery threatens such established regulatory mechanisms, the argument of Douglas is that such pollution beliefs may very well serve the function of upholding and maintaining the existing social organization of the society. In later work, insights into the relationship between cultural beliefs and the social organization of a group has been applied to “modern societies” to explain differences in people's risk perceptions (see e.g., Douglas & Wildavsky, 1983).

**Figure 5:**
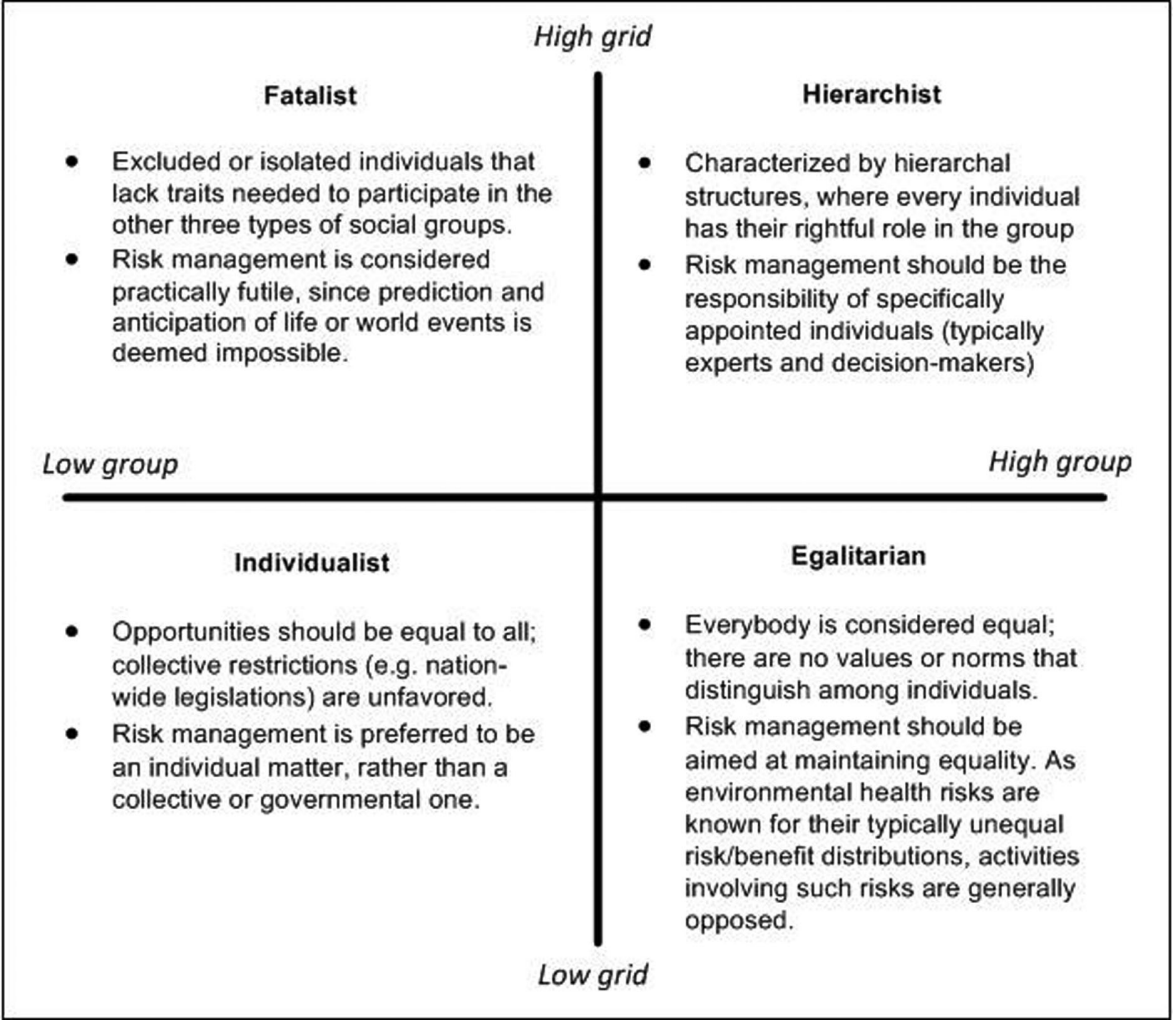
Cultural Theory of Risk's grid–group typology, using the “grid” and “group” concepts as the *y*‐ and *x*‐axis, respectively.

From a systematic analysis of earlier anthropological work, Douglas (1970) discerned two dimensions of “social organization”: *group* and *grid*. *Group* describes the degree an individual is incorporated within a bounded social unit, whereas g*rid* describes the degree of social prescriptions and externally imposed rules, such as behavioral rules and established hierarchies among group members. The resulting “grid–group” typology enables conceptual characterizations of the “social organization” of a group because the typology provides four ideal‐typical “stable types of transactions between people (Hoppe, 2007, p. 290)”: hierarchal groups (high group, high grid), individualist groups (low group, low grid), egalitarian groups (high group, low grid), and fatalist groups (low group, high grid) (see also Fig. 5).

**Figure 6:**
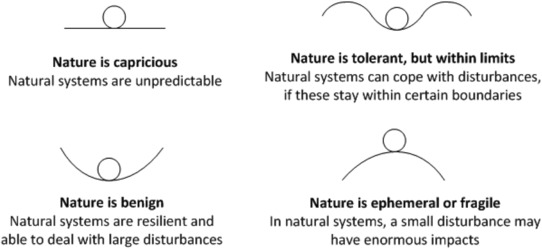
Cultural Theory of Risk's myth of nature typology, represented by a landscape (the type of natural or human physiological system) and a ball (behavior associated with the risk).

Besides the anthropological‐based grid–group typology, Schwartz and Thompson (1990) argue that a complementary typology can be derived from insights from the field of ecology. This typology, consisting of four “myths of nature,” addresses four different perspectives of “ecosystems stability.” Specifically, Dake (1992) defines a myth of nature as “one set of beliefs about what the world is like, what its risks are like, and who is to blame for untoward events” (p. 24). The four myths of nature are “nature is capricious,” “nature is benign,” “nature is tolerant, but within limits,” and “nature is ephemeral or fragile” (see Fig. 6). The key idea is that all four perspectives of ecological stability are technically legitimate, but each proponent of a particular myth of nature will typically view the other myths of nature as irrational (Schwartz & Thompson, 1990). However, some authors problematize the actual degree of correspondence of each of the four myths of nature with one of the four ways of life (see e.g., Grendstad & Selle, 2000).

From the point of view of CTR, regulatory differences can occur due to differences in the cultural beliefs held by influential actors, resulting from differences in these actors' social environment and the organization thereof. The grid–group and myths of nature typologies can function as (separate) concrete yardsticks to discriminate among actors' beliefs about human and physical nature, respectively. For example, we would expect that actors holding individualist beliefs would advocate policies that appeal to these beliefs. Because investments in automobile infrastructure would enhance personal freedom of mobility, we would expect individualists to support such risk management measures. By contrast, the associated increased vehicle capacity of roads would benefit the car users themselves, but could negatively affect nearby residents through increased sound and air pollution. Because this would involve unequal distributions of risks and benefits, thereby undermining egalitarian beliefs, we would expect egalitarian groups to oppose such management measures.

#### 3.2.5. Participatory Approaches to Risk Assessment and Risk Management

The RAP has proven an indispensable tool to support evidence‐based decision making. However, a string of controversies over the assessment of high‐profile chemical and physical risks has unveiled some of its vulnerabilities (see e.g., Jasanoff, 1990). From the 1990s onward, the U.S.‐based National Research Council published several reports on how risk assessments should remain credible and authoritative in times of scientific uncertainty and strong competing interests. In 2005, the “European” International Risk Governance Council published its white paper, which addressed similar challenges.

**Figure 7:**
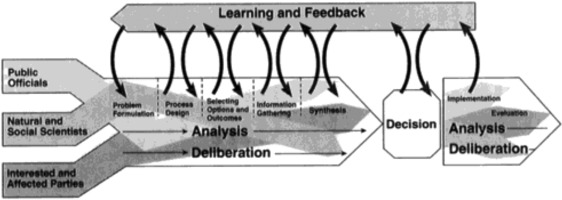
The analytic–deliberative process as proposed in the NRC's 1996 “Understanding Risk” report. *Source*: NRC, 1996. Permission to reprint this figure has been obtained from the copyright holder (U.S. National Academies Press).

In the report “Understanding Risk” of the NRC (1996), the main recommendation was to fundamentally reconsider the concept of “risk characterization.” Instead of viewing risk characterization as a summary or translation of mainly biomedical risk knowledge, a much broader conceptualization of risk characterization was envisioned. In particular, risk characterizations should be decision‐driven, rather than predominantly science‐driven, activities. The multifaceted nature of risk should be acknowledged, extending the scope of risk characterization to include risks relevant to interested and affected parties, such as risks to economic well‐being and the potential to undermine personal, social, cultural, and ethical values. Subsequently, the problem formulations, concerns, needs, and interests of interested and affected parties should continuously be taken into account and merged into an “analytic–deliberative” process of risk assessment and risk management (see Fig. 7). Analytic, since decisions should be informed by rigorous, state‐of‐the‐art science, and deliberative, since interested and affected parties should participate in all phases of the risk assessment process.

Subsequently, in the report “Science and Decisions” of the NRC (2009), the National Research Council revisited the position of risk assessment in contemporary decision making. This volume mainly discusses how the process of risk analysis can be shaped in such a way that risk assessment outcomes provide maximum utility to risk management officials. This time, the 1983 version of the RAP reemerged, but it was explicitly placed in the broader context of political and societal decision‐making processes. The NRC recommends to collectively develop problem definitions and an overview of reasonably foreseeably risk management options before risk assessments are formally planned and conducted. Accordingly, actors should be involved before, during, and after the risk assessment by using systematic participatory processes, similar to the recommendations of the NRC's 1996 report. For an overview of this detailed framework, please see NRC (2009).

**Figure 8:**
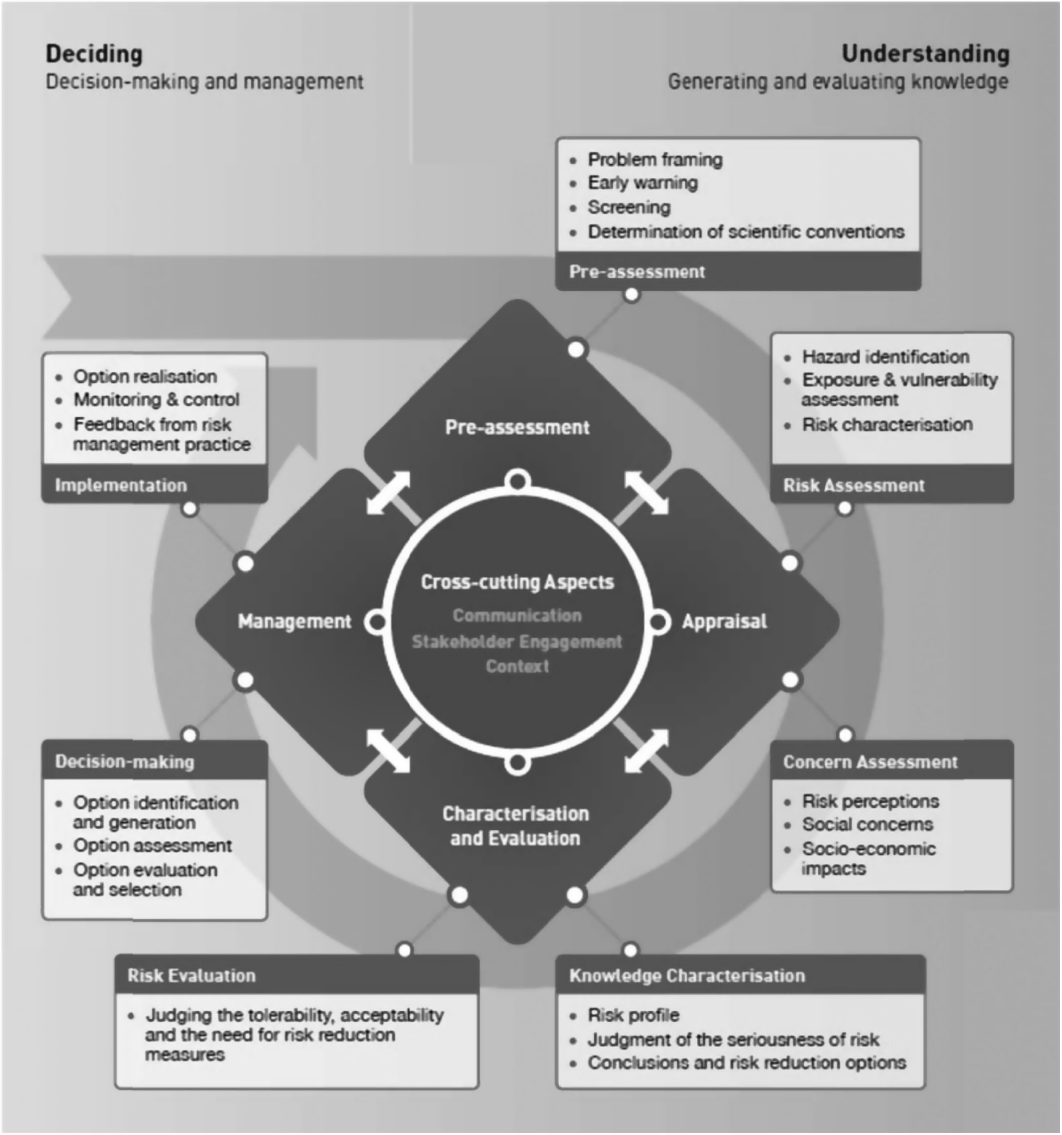
The Risk Governance Framework as proposed by the IRGC. *Source*: IRGC, 2017. Permission to reprint this figure is automatically granted by the copyright holder (EPFL International Risk Governance Center) under condition of acknowledgment.

In Europe, similar calls for the participation of interested and affected parties in risk management were made by both governmental (SCHER/SCENIHR/SCCS, 2013) and independent scientific bodies (IRGC, 2005). Notably, the International Risk Governance Council (IRGC) developed a procedural framework that outlines an inclusive approach to the governance of risks. The framework includes a “risk handling chain” consisting of four phases: preassessment, risk appraisal, tolerability & acceptability judgment, and risk management (IRGC, 2005). The IRGC framework (see also Fig. 8) views analytical risk research and risk perception research as complementary. Among others, this follows from the “risk appraisal” phase, which includes both a technical risk assessment and a concern assessment. In addition, responsible decision making on systemic risks requires appropriately designed and systematic actor participation, in accordance with the degree of complexity, uncertainty, and ambiguity that characterizes the risk‐related knowledge (Hermans, Fox, & van Asselt, 2012; IRGC, 2005, 2008; Renn, 2008; van Asselt & Renn, 2011). According to Renn (2008), “complexity” should here be understood as the difficulty to identify and quantify causal relationships between a variety of potential hazards and the multitude of potential effects following exposure. “Uncertainty” pertains to a situation where the type or nature of any adverse effects, or the likelihood of these effects, cannot be described precisely. Finally, ambiguity refers to a situation where several legitimate and meaningful interpretations of accepted risk assessment results coexist. Nanotechnology risks (IRGC, 2006) and risks posed by synthetic biology (IRGC, 2009) are examples of risks that have been analyzed using a risk governance perspective.

In sum, risk management in democratic societies requires the involvement of interested and affected parties in the various steps of risk assessment and risk management, and explicit and continuous attention to their risk perceptions, including concerns, interests, and needs. The analytical approach to risk is still considered indispensable, though it is seen as one of the necessary ingredients for sound risk management. From the point of view of these frameworks, regulatory differences could occur due to differences in the degree of inclusion of interested and affected parties in risk management and the range of risk perceptions, concerns, interests, and needs voiced by these parties included within risk management.

#### 3.2.6. Advocacy Coalition Framework

According to the founders of the Advocacy Coalition Framework (ACF), Sabatier and Jenkins‐Smith (1993), the ACF provides a theoretical lens to investigate complex public policy questions. The principal aim of the ACF is to simplify the complexity of public policy for research purposes because public policy issues typically include many different actors with different beliefs and interests, uneven power relations between these actors, and uncertain scientific knowledge (Weible, Sabatier, & McQueen, 2009).

**Figure 9:**
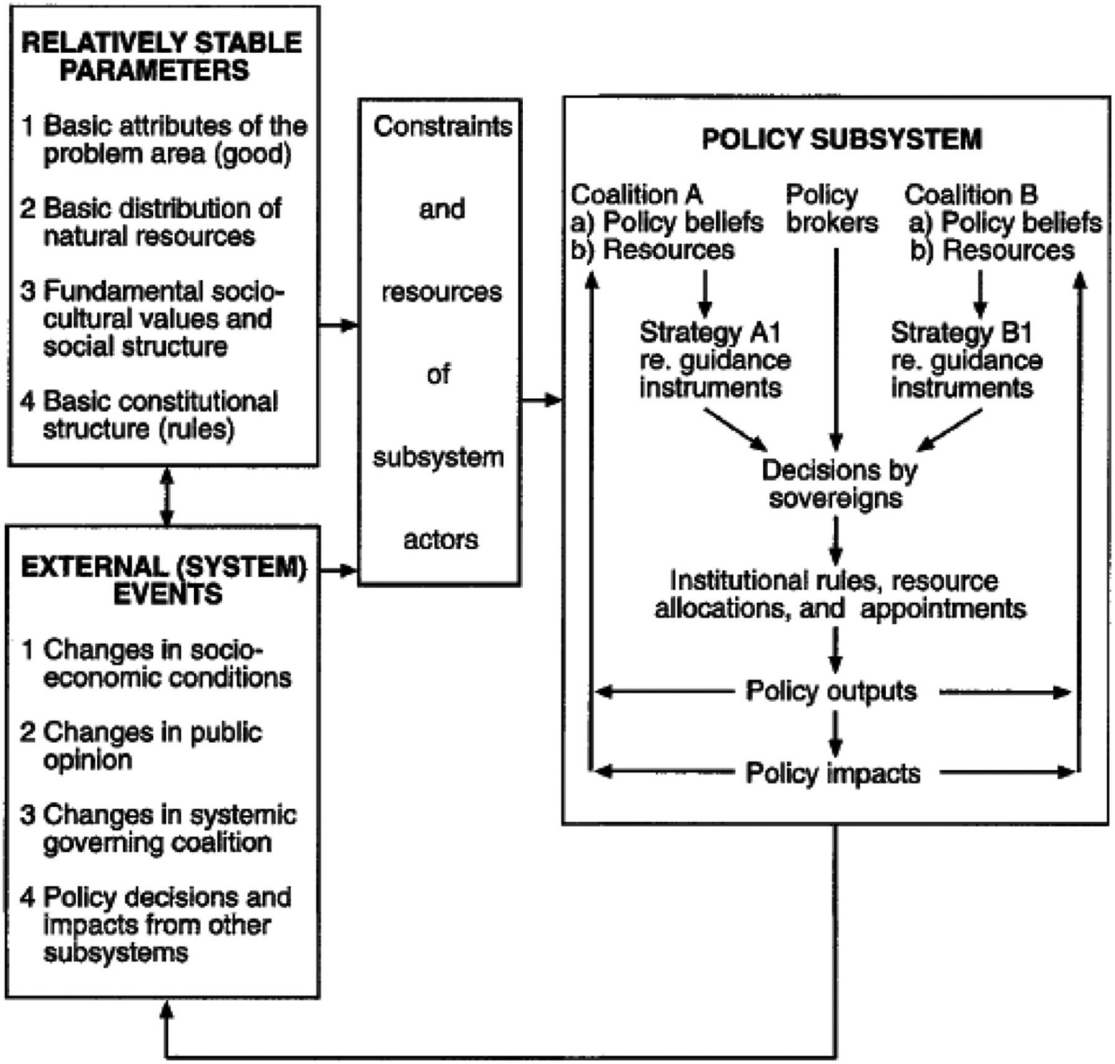
The Advocacy Coalition Framework. *Source*: Sabatier, 1998. Permission to reprint this figure has been granted by the copyright holder (Taylor & Francis).

The ACF itself is a comprehensive framework (see also Fig. 9), with a wide variety of proposed causal relationships, several testable hypotheses, and a set of underlying assumptions. For reasons of brevity, we will focus on two essential concepts of the ACF: the *policy belief system* and the *advocacy coalition*.

**Figure 10:**
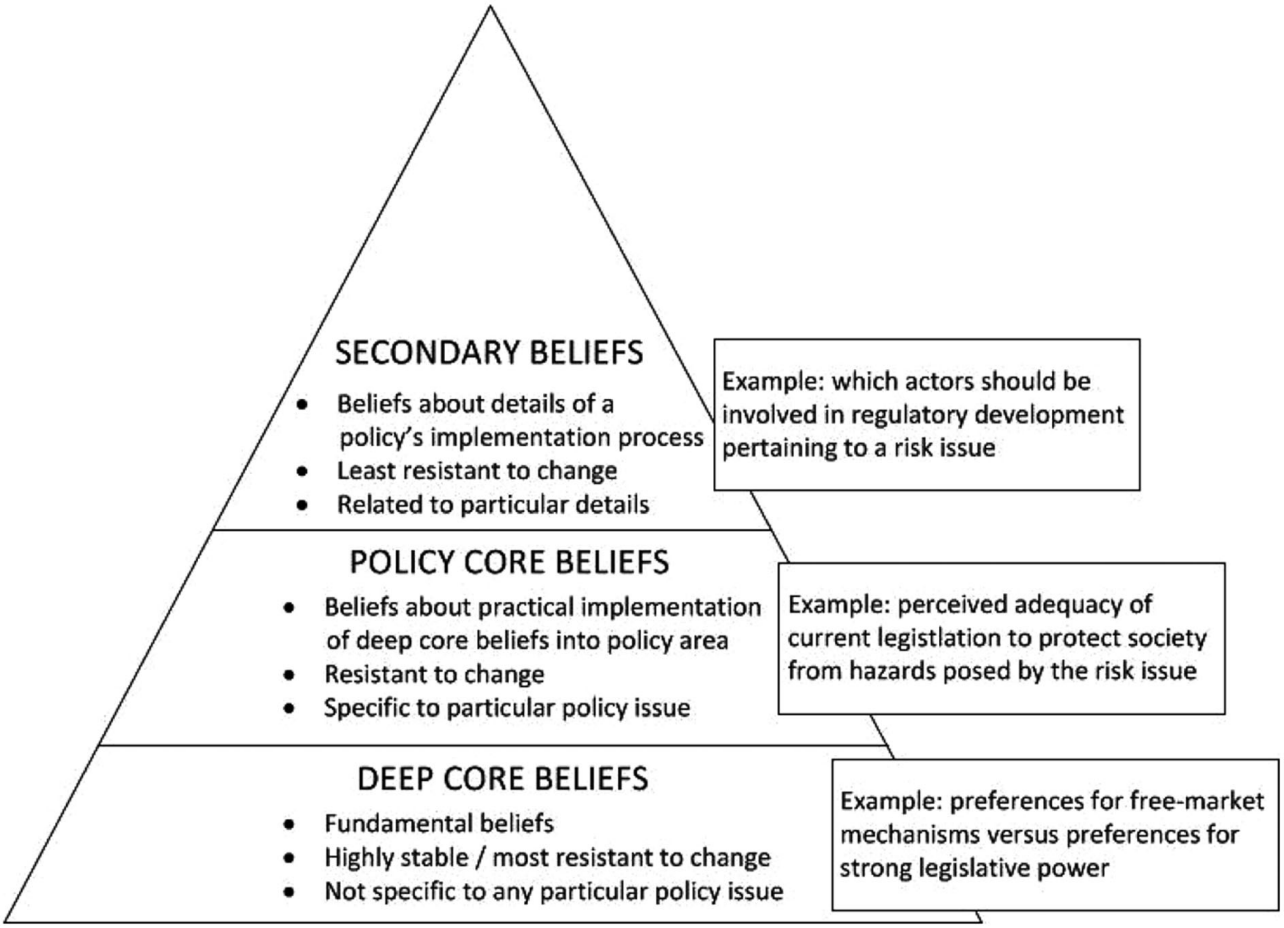
The three layers of beliefs, including examples, which together form a hierarchal policy belief system.

A policy belief system is a three‐tiered, hierarchal system of beliefs held by those actors that deal with the policy issue on a professional basis (i.e., “policy elites”) (Sabatier & Jenkins‐Smith, 1993). The policy belief system is a key element of the ACF because the ACF explicitly identifies the beliefs held by politically active actors as the driving force for their political behavior (Weible et al., 2009). A policy elite's policy belief system consists of broad and fundamental deep core beliefs, issue‐specific policy core beliefs, and highly specific, more instrumental secondary beliefs (see Fig. 10).

Though each individual actor is assumed to possess a policy belief system, actors often share certain beliefs. The ACF hypothesizes that when actors share policy core beliefs, they will be inclined to form an advocacy coalition. Here, the term “coalition” should be understood as a circumstantial, loosely connected group of stakeholders that cooperate to a certain degree to have their preferred policy objective implemented. In practice, various advocacy coalitions with competing policy preferences will coexist.

Originally, the ACF was put forward as an alternative to a dominant earlier theory in (public) policy research, the Policy Cycle (also known as the “stages heuristic”; see e.g., Jann & Wegrich, 2007). The ACF may be considered part of the (public) policy studies tradition, whereas the framework also draws, on differing levels, from other disciplines. Prime examples are several theories and broader insights derived from psychology, such as the theory of reasoned action and cognitive dissonance theory (Sabatier, 1998; Sabatier & Jenkins‐Smith, 1993). The ACF has been used to analyze various national and international policies, such as financial/economic policies, social policies and education policies, and (public) health and environmental policies, such as air pollution policies and tobacco/smoking policies (Jenkins‐Smith, Nohrstedt, Weible, & Sabatier, 2014; Sabatier, 1998; Sabatier & Jenkins‐Smith, 1993; Weible & Sabatier, 2007).

From the point of view of the ACF, regulatory differences could occur through the prominence of different advocacy coalitions within different countries for the same policy area. Identifying the deep core beliefs, policy core beliefs, and relevant secondary beliefs of the key actors will then be instrumental in discerning these advocacy coalitions.

#### 3.2.7. Social Amplification of Risk Framework

**Figure 11:**
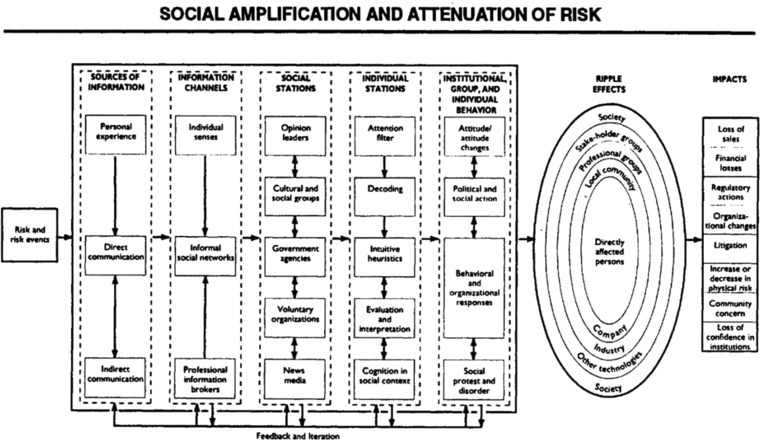
The Social Amplification of Risk Framework. *Source*: Kasperson et al., 1988. Permission to reprint this figure has been granted by the copyright holder (John Wiley and Sons).

The concept of social amplification of risk was outlined by Kasperson and colleagues in their Social Amplification of Risk Framework (SARF). SARF sets out to explain why a risk associated to relatively limited hazards, exposure, or adverse effects may evoke strong societal reactions, or vice versa, why risks with a substantial attributed impact (from a technical risk assessment perspective) may be attenuated in society (Kasperson et al., 1988; Kasperson, Kasperson, Pidgeon, & Slovic, 2003). SARF introduces the phenomenon of “social amplification of risk” to explain how risk information is continuously framed and reframed by various societal actors, leading to an increased (amplified) or decreased (attenuated) societal response to the risk (see Fig. 11). For example, Frewer, Miles, and Marsh (2002) noted that increases of media attention to the risks of genetically modified (GM) foods, sparked by worrisome though unpublished research findings, resulted in increased perceptions of risk and decreased perceptions of benefits of GM foods. When the amount of media reporting on GM foods decreased through time, the occurred amplification similarly appeared to diminish, though perception of benefits of GM foods remained depressed for at least a year after the initial media reporting.

In terms of its disciplinary orientation, SARF is explicitly described as an interdisciplinary framework that aims to integrate several strands of previously isolated risk‐oriented research (Kasperson et al., 1988). Accordingly, a key feature is the integration of the technical perspective of risk with psychological, sociological, and cultural perspectives of risk. In turn, the framework tries to reconcile the psychological understanding of risk that is central to the Psychometric Paradigm with the sociological and anthropological understanding of risk developed within the Cultural Theory of Risk (Kasperson et al., 1988; Tansey & O'Riordan, 1999). However, some scholars have problematized the attempt to reconcile these two perspectives of risk (see, e.g., Rayner, 1992).

The SARF may be relevant to environmental health risk management because SARF proposes that risk managers or other decisionmakers may react very strongly, or suddenly change their management strategy, as a result of amplified societal reactions. For example, in the Brent Spar controversy, Shell changed its disposal strategy from deep sea disposal to the allegedly less favorable on‐shore dismantling after intense societal uproar, mobilized by Greenpeace (Bakir, 2005; Löfstedt & Renn, 1997). The phenomenon of excessive political reactions in response to amplification processes is similar to the notion of “risk regulation reflex,” a topic of research in Dutch political science literature. Stringent, sometimes highly invasive measures may be implemented that go substantially beyond the direct effects involved, due to or in anticipation of public responses following the risk event (WRR, 2012). Then, differences in the occurrence of social amplification and the “risk regulation reflex” among countries may explain international differences in risk management. In country A, a certain risk frame may have resonated sufficiently to produce social amplification, whereas in country B, the risk may have passed by relatively unnoticed (i.e., no or little “risk selection”). In country A, subsequent public pressures to act may cause policymakers to revise current risk management or act in accordance with the “risk regulation reflex,” whereas such societal and political dynamics will have eluded country B.

#### 3.2.8. Hofstede's Model of National Cultures

Hofstede's Model of National Cultures (HMNC) mainly includes six dimensions of national culture, mostly developed from studies into the corporate cultures of subsidiaries of a multinational high‐technology company referred to as HERMES (Hofstede, 1980). In the period between 1967 and 1973, Hofstede set out multiple attitude surveys in HERMES subsidiaries in a wide variety of countries (maximum: 67 countries). In general, these surveys included four types of questions, to be answered by the subsidiaries' employees: questions pertaining to employee *satisfaction* (e.g., satisfaction with a certain job aspect), questions asking for employee *perceptions* (e.g., perceived level of stress), questions pertaining to *personal goals and beliefs* (e.g., preferred type of manager), and *demographics* (e.g., years of education). Subsequently, a subset of these questions proved useful in distinguishing four dimensions of national culture: power distance, uncertainty avoidance, individualism, and masculinity. Two dimensions were added in a later stage: long‐term orientation (Hofstede, 1991) and indulgence (Hofstede, Hofstede, & Minkov, 2010) (see also Table I).

**Table I:** Overview of the Six Dimensions of National Cultures Developed by Hofstede and Colleagues, Including Related Core Values and Definitions

Cultural Dimension	Related Core Value (After Hofstede, 2011)	Definition
Power distance (high vs. low)	How a society's members find solutions to the fundamental issue of human inequality	“[T]he extent to which the less powerful members of institutions and organizations within a country expect and accept that power is distributed unequally” (Hofstede & Hofstede, 2005, p. 46).
Uncertainty avoidance (high vs. low)	How stressful the idea of an unknown future is to a society's members	“[T]he extent to which the members of a culture feel threatened by ambiguous or unknown situations” (Hofstede & Hofstede, 2005, p. 167).
Individualism (vs. collectivism)	How a society's individual members integrate into certain groups	Individualism: a society “in which the ties between individuals are loose: every one is expected to look after himself … and his … immediate family” (Hofstede & Hofstede, 2005, p. 76). Collectivism: a society “in which people from birth onward are integrated into strong, cohesive in‐groups, which throughout people's lifetimes continue to protect them in exchange for unquestioning loyalty” (Hofstede & Hofstede, 2005, p. 76).
Masculinity (vs. femininity)	How the division of emotional roles of men and women has taken shape in a society	Masculine society: “emotional gender roles are clearly distinct: men are supposed to be assertive, tough, and focused on material success, whereas women are supposed to be more modest, tender and concerned with the quality of life” (Hofstede & Hofstede, 2005, p. 120). Feminine society: “emotional gender roles overlap: both men and women are supposed to be modest, tender, and concerned with the quality of life” (Hofstede & Hofstede, 2005, p. 120).
Long‐term orientation (vs. short‐term orientation)	How a society's members direct their efforts timewise	Long‐term orientation: “fostering of virtues oriented toward future rewards; in particular, perseverance and thrift” (Hofstede & Hofstede, 2005, p. 210). Short‐term orientation: “fostering of virtues related to the past and present; in particular respect for tradition, preservation of ‘face’ and fulfilling social obligations” (Hofstede & Hofstede, 2005, p. 210).
Indulgence (vs. restraint)	How a society's members go about satisfying their wants and needs to enjoy life	Indulgent society: “allows relatively free gratification of basic and natural human desires related to enjoying life and having fun” (Hofstede, 2011, p. 15) Restrained society: “controls gratification of needs and regulates it by means of strict social norms” (Hofstede, 2011, p. 15).

From the point of view of HMNC, regulatory differences can occur due to differences in national culture, primarily through differences in the characterization of countries' cultures using Hofstede's six dimensions. For example, a more “masculine” country would value entrepreneurship, competitiveness, and financial prosperity and would subsequently emphasize the opportunities offered by potentially risky technological development. We would expect that, in such cultures, general hesitance occurs toward innovation‐inhibiting precautionary interventions. Alternatively, a more “feminine” country would emphasize the well‐being of the collective, and particularly that of vulnerable groups. In order to inherently protect all populations from potentially hazardous exposures following from a risky technological development, we would expect that such cultures generally prefer (early) precautionary action over “laissez faire” policies.

### 3.3. Key Ideas and Questions Drawn from the Conceptual Frameworks

The eight conceptual frameworks discussed in this article draw attention to different aspects of risk management processes. The second column of Table II provides an overview of the key ideas introduced by the various frameworks. From these ideas, we derived questions that could guide further empirical work in the form of interviews and document analyses (see the third column of Table II). Whether these questions will actually help understand why certain risk management strategies have been selected, and why such strategies differ between countries, will follow from future empirical research.

**Table II:** The Eight Conceptual Frameworks and Their Properties to Explain Variation in International Risk Management Strategies, Including Questions Derived from These Frameworks that Could be Used to Empirically Study These Variations; The Questions Pertaining to Hofstede's Model of National Culture are Presented in Italic Because These Questions Cannot Be Used Directly During Interviews

Name and (Disciplinary) Orientation of Conceptual Frameworks	Key Ideas Brought Forward by the Frameworks	Questions Relevant to the Analysis of International Differences in Environmental Health Risk Management
Risk Assessment Paradigm (RAP); analytical perspective used by expert risk assessors	Preferably quantitative assessment of risk numbers, based on toxicological working mechanisms, dose–response knowledge, and exposure in the population Separation of risk assessment (life science oriented) and risk management (normative, policy‐oriented balancing of various different perspectives) Often interpretative ambiguity about hazard and risk science base in risk assessment	How was risk assessment knowledge weighted against other perspectives? Was uncertainty addressed, and how did uncertainties affect risk management? Were safety or assessment factors used to define safe levels? Were availability and risks of alternatives considered? Was precaution addressed explicitly?
Advisory roles of experts in risk management processes (ARERMP); interdisciplinary field of research dealing with roles of subject‐matter experts in risk management processes	Experts can take different roles when providing advice to policymakers; ideal typical expert roles include pure scientist, science arbiter, issue advocate, and honest broker of policy alternatives Personal views of experts affect their willingness to advise on different action perspectives; ideal‐typical expert roles include science absolutist, technological optimist, environmental centrist, cautious environmentalist, environmental absolutist Expert roles are strongly context dependent, depending on, e.g., the characteristics of the issue and the organization the expert works in	Which expert roles can be identified in the risk management process? Were specific expert roles dominant? How could the occurrence of these roles have affected risk management process? Would inclusion of other expert roles have allowed for other action perspectives? To what extent were different expert roles present in the expert commission or decision‐making team?
Psychometric Paradigm (PP); psychological perspective on risk perception of the general public	Evaluations of risks depend on how individuals construct risks Focus on acceptability of risk by individuals, not on risk numbers applying uniformly to any individual Several dimensions of risk acceptability defined, e.g., involuntary nature of risk, equitable distribution of risks and benefits, perceived controllability Actors use heuristics in the evaluation of information and of risks	How does the general public perceive the risk? Could these perceptions be characterized as “dread risk” or “unknown risk”? How may these risk perceptions have impacted risk management?
Cultural Theory of Risk (CTR); sociological and anthropological perspective on risk perception of lay people and culturally determined (core) beliefs	Characterization of two dimensions of social organization, i.e., grid and group; four ways of life follow from different types of social organization: egalitarian, individualist, fatalist, hierarchist; these four views characterize attitudes to acceptability of risks and preferred management of risks Four different perspectives on “ecological stability,” or myths of nature: nature is benign, nature is ephemeral or fragile, nature is robust, but within limits, nature is capricious; these four beliefs characterize attitudes to acceptability of risks and preferred management or risk A relationship between the four ways of life and the four myths of nature is hypothesized	Do different groups hold different perceptions of risk? To what extent did arguments in the risk management process resemble any way of life? To what extent did arguments in the risk management process resemble any myth of nature? Were one of these perspectives dominant in the decision‐making discourse? How could this have affected the decisions made? Did the composition of the expert commission or decision‐making team sufficiently cover the different ways of life and myths of nature perspectives?
Participatory approaches to risk assessment and management (PARAM); procedural frameworks combining the analytical approach to risk assessment with insight from “social scientific” risk perception research	Stakeholder involvement in risk assessment, management and governance Concern assessment parallel to risk assessment (IRGC framework) Decision contexts and societal needs should be the starting point for assessments and characterizations of risk	Which actors have been involved in the risk management process? To what extent have perceptions of risk been taken forward in the decision‐making process? What specific policy or societal questions were the risk assessments required to address?
Advocacy Coalition Framework (ACF): perspective from policy sciences	Beliefs held by (professional) actors can be structured in a hierarchal, three‐layered policy belief system, consisting of deep core beliefs, policy core beliefs, and secondary beliefs Actors holding similar policy core beliefs will cooperate in advocacy coalitions to have their preferred policy measures implemented Various advocacy coalitions will typically coexist and attempt to advance their political agenda	What are the policy belief systems of the most influential actors? What advocacy coalitions have been present in the decision‐making process? Has a certain advocacy coalition been dominant in the decision‐making process? What were the most important (core) beliefs of the actors cooperating in the dominant advocacy coalition?
Social Amplification of Risk Framework (SARF); integrative perspective drawing from multiple fields of risk research	Information processing and interactions between different actors determine the extent of social amplification or attenuation of risks Actors emphasize different parts of messages, leading to different framings and different evaluations of information and of risks	Did certain risk frames resonate with particular actors, including members of the general public? Did this resonance induce amplification or attenuation? To what extent may this amplification have affected the decision‐making process?
Hofstede's Model of National Culture (HMNC); perspective focusing on how cultures differ between countries	The culture of individual countries can be characterized in terms of six dimensions: power distance, uncertainty avoidance, individualism, masculinity, long‐term orientation, indulgence	*How does the country score on the six dimensions of Hofstede?* *Are these differences between the analyzed countries, with respect to the scoring on certain dimensions?* *To what extent do potential differences in scoring relate to the observed differences in risk management strategies?*

## 4. DISCUSSION AND CONCLUSIONS

In this article, we have explored a range of theoretical perspectives applicable to the analysis of reasoning behind divergent risk management strategies in the environmental health domain. This exploration was performed while explicitly acknowledging the often complex and intimate relationships between science and policy. Ultimately, our aim is to identify, understand, and compare the arguments used to develop national‐level decision making in various countries, for the same risk, and how, and to what extent scientific expertise is involved in shaping these arguments.

### 4.1. Overlap and Differences in Disciplinary Orientations

When comparing the disciplinary orientations of the eight conceptual frameworks (using the first column of Table II), both differences and overlap can be observed. To gain more insight in the areas of science in which the frameworks are used, and how these areas contrast or match with one another, we conducted a literature search in Scopus. For each conceptual framework, we compiled a list of relevant publications using a specific search query (see Table III). We then categorized the resulting list using the 28 subject areas (including “undefined”) contained in Scopus and calculated the following ratio for each subject area: the number of publications falling within 1 of the 28 subject areas as compared to the total number of publications found for that specific framework. Table III shows these ratios, as well as the list of queries used and the 28 subject areas used for categorization. We arbitrarily consider a subject area significant for a particular conceptual framework when at least 5% of the total literature was found to fall into that area of science.

**Table III:** Upper Part: Results from the Literature Search Showing the Percentage of Publications Falling into a Particular Subject Area, as Compared to the Total Number of Publications Gathered for that Conceptual Framework; Percentages Printed in **Bold**: Assumed Significance (Percentage ≥5%); Subject Areas Printed in **Bold**: at Least Six of the Eight Frameworks Score ≥5% (Assumed Significance) for a Particular Subject Area; Lower Part: List of the Abbrevations Used in the Upper Part of Table III and the Queries Used to Perform the Literature Search in Scopus

	Conceptual Frameworks							
Subject Areas Used in Scopus	RAP	ARERMP	PP	CTR	IRGC	ACF	SARF	HMNC
Agricultural and Biological Sciences	**5%**	**5%**	3%	0%	4%	**5%**	1%	0%
Arts and Humanities	0%	**9%**	2%	4%	2%	2%	2%	4%
Biochemistry, Genetics, and Molecular Biology	**6%**	3%	1%	0%	1%	0%	1%	0%
**Business, Management, and Accounting**	0%	2%	**7%**	**11%**	**9%**	**6%**	**11%**	**34%**
Chemical Engineering	3%	1%	0%	0%	3%	1%	0%	0%
Chemistry	3%	0%	0%	0%	4%	0%	0%	0%
Computer Science	0%	1%	3%	1%	0%	0%	1%	**11%**
Decision Sciences	0%	0%	1%	2%	1%	0%	1%	4%
Dentistry	0%	0%	0%	0%	0%	0%	0%	0%
Earth and Planetary Sciences	0%	**5%**	3%	3%	**5%**	1%	3%	0%
Economics, Econometrics, and Finance	0%	3%	1%	2%	4%	4%	3%	**14%**
Energy	0%	1%	2%	0%	1%	1%	1%	1%
**Engineering**	**6%**	**5%**	**19%**	**9%**	**19%**	1%	**17%**	4%
**Environmental Science**	**29%**	**14%**	**12%**	**10%**	**10%**	**24%**	**9%**	1%
Health Professions	0%	1%	0%	1%	0%	0%	0%	0%
Immunology and Microbiology	2%	1%	0%	0%	1%	0%	0%	0%
Materials Science	1%	0%	1%	0%	2%	0%	0%	0%
Mathematics	0%	1%	1%	0%	2%	0%	1%	1%
**Medicine**	**19%**	**15%**	**15%**	**14%**	**6%**	**5%**	**17%**	1%
Multidisciplinary	0%	1%	0%	0%	0%	0%	0%	0%
Neuroscience	0%	0%	0%	0%	0%	0%	0%	0%
Nursing	0%	3%	1%	1%	0%	1%	0%	0%
Pharmacology, Toxicology, and Pharmaceutics	**22%**	1%	1%	0%	1%	0%	0%	0%
Physics and Astronomy	0%	1%	0%	0%	2%	0%	0%	0%
Psychology	0%	1%	**7%**	**7%**	0%	1%	**7%**	4%
**Social Sciences**	3%	**26%**	**16%**	**31%**	**22%**	**48%**	**23%**	**19%**
Veterinary	0%	1%	0%	0%	1%	0%	0%	0%
Undefined	0%	0%	0%	0%	0%	0%	0%	0%
**Abbreviations used**	**Query used in Scopus**							
RAP: Risk Assessment Paradigm	TITLE‐ABS‐KEY(“risk assessment paradigm” AND							
	(“hazard” OR “exposure”))							
ARERMP: Advisory roles of experts in risk management processes	TITLE‐ABS‐KEY(“Policy” AND “Scien*” AND Expert*							
	AND “Role*” AND “Advi*”)							
PP: Psychometric Paradigm	TITLE‐ABS‐KEY(“Psychometric Paradigm” AND “Risk”)							
CTR: Cultural Theory of Risk	TITLE‐ABS‐KEY(“Cultural Theory” AND “Risk”)							
IRGC: IRGC Risk Governance Framework	TITLE‐ABS‐KEY(“International Risk Governance							
	Council” OR “Risk Governance Framework”)							
ACF: Advocacy Coalition Framework	TITLE‐ABS‐KEY(“Advocacy Coalition Framework”)							
SARF: Social Amplification of Risk Framework	TITLE‐ABS‐KEY(“Social Amplification” AND “Risk”)							
HMNC: Hofstede's Model of National Cultures	TITLE‐ABS‐KEY(“Hofstede” AND “National Culture”)							

Most notably, we observe that the RAP and HMNC are applied in distinctly different areas of science as compared to the other conceptual frameworks. Table III shows that the RAP stands out by being the only framework where we found a significant share of literature related to “pharmacology, toxicology, and pharmaceutics,” but lacking a significant share of literature related to “social sciences.” These findings reinforce the idea that literature referencing the RAP still has a particularly technical and biomedical disciplinary orientation. HMNC stands out by its strong orientation toward “business, management, and accounting” literature, by being the only framework with a significant share of literature in “computer science” and “economics, econometrics and finance,” and by missing a significant share of literature for “environmental science” and “medicine.” These findings may be unsurprising because HMNC has no substantive links to any type of environmental health‐related risk research. Alternatively, the six other frameworks all have significant shares of literature in both the “social sciences” and some of the biomedical‐related (“environmental science” and “medicine”) areas of science. These findings appear to reinforce the idea that the majority of frameworks included in this article combine social scientific perspectives with case studies related to human or environmental risks. Concluding, this bibliometric comparison does appear to underline the “polythetic” nature of the concept of “risk.”

In terms of limitations, we used one single search engine (i.e., Scopus), we used relatively simple search queries, and we did not exclude any publications from the list of literature obtained using the query. However, we think that our pragmatic approach to a literature search is appropriate to get a taste of the areas of science relevant to each of the eight conceptual frameworks.

### 4.2. Visualization of (Conceptual) Relationships Between the Frameworks

**Figure 12:**
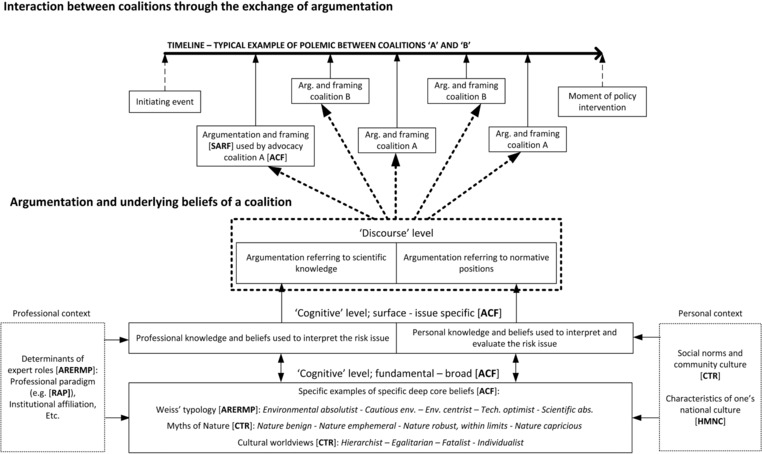
An overarching framework that visualizes the various (conceptual) relationships between the eight conceptual frameworks. Abbreviations of the names of the frameworks, printed in [**BOLD CAPITALS**] and in between square brackets, are used to signal that an idea or concept of that respective framework has been used as a source of inspiration for that specific section of the overarching framework. List of the abbreviations used: [**SARF**], Social Amplification of Risk Framework; [**ACF**], Advocacy Coalition Framework; [**ARERMP**], Advisory Roles of Experts in Risk Management Processes; [**RAP**], Risk Assessment Paradigm; [**CTR**], Cultural Theory of Risk; [**HMNC**], Hofstede's Model of National Cultures.

Although there are distinct disciplinary differences between some of the conceptual frameworks, some conceptual overlap can be found between many of them. For example, the four worldviews in the grid/group typology of the CTR could provide a theory‐based specification of the fundamental “deep core beliefs” in the ACF (Hoppe, 2007; Jenkins‐Smith, Silva, Gupta, & Ripberger, 2014). Our attempt to visualize as many connections as possible between the key concepts of the various conceptual frameworks resulted in one overarching framework (see Fig. 12).

In short, we propose two levels of analysis: a level of interaction occurring *between* advocacy coalitions, and a level of argumentative and cognitive processes taking place *within* the stakeholder or coalition. The upper level (upper part of Fig. 12) is mostly inspired by the SARF and the ACF and consists of a timeline, in which the competing advocacy coalitions make their argumentative moves to influence the political course of action. The advocacy coalition whose arguments (and framings contained therein) amplify most strongly in society will be expected to dictate this course of action. Next, we believe that an advocacy coalition's argumentative moves are performed in accordance with predetermined sets of beliefs (bottom part of Fig. 12). We draw from the ACF to make a distinction between *surface* beliefs that are specific to the risk, and *fundamental* beliefs that transcend specific issues. We identify specific types of fundamental beliefs: the five expert roles developed by Weiss (2003) and the four myths of nature and four cultural worldviews developed within the framework of the CTR. Next, we distinguish scientific/professional reasoning and knowledge from normative reasoning and personally held beliefs. We propose that professional issue‐specific knowledge and beliefs are influenced by the various determinants of expert roles outlined by Spruijt et al. (2014). By contrast, personal knowledge and beliefs are influenced by social norms, community culture (cf. CTR), and the characteristics of one's national culture (cf. HMNC).

In terms of limitations, some connections between frameworks may have been missed or may have been overinterpreted, and this framework is not supported by empirical evidence. However, the framework does provide, in one relatively simple figure, a visualization of the various concepts and ideas that may be relevant for the study of divergent risk management strategies in the environmental health domain.

### 4.3. Strengths and Limitations

As far as we are aware, this article is the first study where conceptual frameworks from a wide variety of academic disciplines are applied to seek explanations for differences in environmental health risk management strategies between countries. We argue that these conceptual frameworks are complementary to one another, by offering different concepts and ideas to study these differences. Indeed, Slovic (1999) wrote that “whoever controls the definition of risk controls the rational solution to the problem at hand” (p. 689), thereby drawing an explicit link between the numerous perspectives of risk and how these impact what is considered to be, and how one should arrive at, “rational” risk management strategies.

In this article, we are mainly interested in differences in environmental health risk management between countries. However, within countries, risk management strategies can also differ from policy area to policy area (see e.g., Hood, Rothstein, & Baldwin, 2001). For example, Johannesson, Hansson, Rudén, and Wingborg (1999) showed that in Sweden, major differences exist between the policy areas of occupational safety and health, environmental protection, and chemicals control, in terms of the organization of legal mandates across the relevant agencies, the regulatory strategies followed and the enforcement activities involved. Although we acknowledge the typical diversity of risk management strategies within a country, we argue that discussions about how and why such *intranational* diversity occurs are beyond the scope of this article.

In addition, we realize that the conceptual frameworks discussed in this article offer inherently simplified representations of the complexity and dynamics associated with risk management processes. A key contextual factor that is not explicitly part of this theoretical review is how preconditions and regulatory boundaries set by existing regulatory frameworks could influence risk management processes. Regulatory frameworks in the environmental health domain typically provide standards (at least partially) based on scientific evidence, and provisions for the generation of specific types of scientific data (minimally) necessary to evaluate whether the potential hazard meets the established safety standards. These arrangements are developed based on negotiations between the interested and affected parties. These parties can strategically use the room offered by the resulting regulatory arrangements to serve their specific interests. For example, various parties prefer to reduce the amount of animal tests (for various reasons), and each party can use the provisions available in a regulatory framework to cater to this preference. A further discussion of such stakeholder maneuvering in the context of particular regulatory frameworks is outside the scope of this review of conceptual frameworks. However, future case studies should take into account the regulatory context, and particularly the associated uses of scientific evidence, in which the risk management process is taking place.

## 5. CONCLUSIONS

We have analyzed a variety of conceptual frameworks in order to develop a list of questions we can use to study divergent (preferred) risk management strategies in the domain of environmental health risks. We were specifically attentive to the role of science in risk management processes. In future research, the analytical value of these questions will be tested empirically through case studies in the field of environmental health risk management.

## References

[risa13165-bib-0001] Aven, T. , & Renn, O. (2010). Risk management and governance: Concepts, guidelines and applications. Berlin Heidelberg: Springer‐Verlag.

[risa13165-bib-0002] Bakir, V. (2005). Greenpeace v. Shell: Media exploitation and the social amplification of risk framework (SARF). Journal of Risk Research, 8(7–8), 679–691. 10.1080/13669870500166898

[risa13165-bib-0003] Beronius, A. , Rudén, C. , Hakansson, H. , & Hanberg, A. (2010). Risk to all or none? A comparative analysis of controversies in the health risk assessment of Bisphenol A. Reproductive Toxicology, 29, 132–146. 10.1016/j.reprotox.2009.11.007 19931376

[risa13165-bib-0004] Covello, V. T. , & Merkhofer, M. W. (1993). Risk assessment methods: Approaches for assessing health and environmental risks. New York: Plenum Press.

[risa13165-bib-0005] Dake, K. (1992). Myths of nature: Culture and the social construction of risk. Journal of Social Issues, 48(4), 21–37. 10.1111/j.1540-4560.1992.tb01943.x

[risa13165-bib-0006] Dolan, T. , Howsam, P. , Parsons, D. J. , & Whelan, M. J. (2013). Is the EU drinking water directive standard for pesticides in drinking water consistent with the precautionary principle? Environmental Science and Technology, 27(10), 4999–5006. 10.1021/es304955g 23590121

[risa13165-bib-0007] Douglas, M. (1966). Purity and danger: An analysis of the concepts of pollution and taboo. London and New York: Routledge.

[risa13165-bib-0008] Douglas, M. (1970). Natural symbols: Explorations in cosmology. London: Cresset Press.

[risa13165-bib-0009] Douglas, M. , & Wildakvsy, A. (1983). Risk and culture: An essay on the selection of technological and environmental dangers. Berkeley and Los Angeles, CA: University of CA Press.

[risa13165-bib-0010] EFSA (European Food Safety Authority) . (2015). Conclusion on the peer review of the pesticide risk assessment of the active substance glyphosate. Parma, Italy: European Food Safety Authority Retrieved from https://www.efsa.europa.eu/en/efsajournal/pub/4302. 10.2903/j.efsa.2023.8164 PMC10369247 37502013

[risa13165-bib-0011] EPA (Environmental Protection Agency) . (2016). *How EPA regulates drinking water contaminants* . Retrieved from https://www.epa.gov/dwregdev/how-epa-regulates-drinking-water-contaminants.

[risa13165-bib-0012] EPA (Environmental Protection Agency) . (n.d.). *The NRC risk assessment paradigm* . Retrieved from https://www.epa.gov/fera/nrc-risk-assessment-paradigm.

[risa13165-bib-0013] FAO/WHO (Food and Agriculture Organization of the United Nations/World Health Organization) . (2016). Pesticide residues in food 2016—Special session of the joint FAO/WHO meeting on pesticide residues. Rome, Italy: Food and Agriculture Organization of the United Nations/World Health Organization Retrieved from https://www.fao.org/3/a-i5693e.pdf.

[risa13165-bib-0014] Fischhoff, B. , Slovic, P. , Lichtenstein, S. , Read, S. , & Combs, B. (1978). How safe is safe enough? A psychometric study of attitudes towards risks and benefits. Policy Sciences, 9(2), 127–152. 10.1007/BF00143739

[risa13165-bib-0015] Frewer, L. J. , Miles, S. , & Marsh, R. (2002). The media and genetically modified foods: Evidence in support of social amplification of risk. Risk Analysis, 22(4), 701–711. 10.1111/0272-4332.00062 12224744

[risa13165-bib-0016] Funtowicz, S. O. , & Ravetz, J. R. (1993). Science for the post‐normal age. Futures, 25(7), 739–755. 10.1016/0016-3287(93)90022-L

[risa13165-bib-0017] Grendstad, G. , & Selle, P. (2000). Cultural myths of human and physical nature: Integrated or separated? Risk Analysis, 20(1), 27–39. 10795336 10.1111/0272-4332.00003

[risa13165-bib-0018] Hermans, M. A. , Fox, T. , & van Asselt, M. B. A. (2012). Risk governance In Roeser S., Hillerbrand R., Sandin P., & Peterson M. (Eds.), Handbook of risk theory. The Netherlands: Springer.

[risa13165-bib-0019] Hofstede, G. (1980). Culture's consequences: International differences in work‐related values. Beverly Hills: Sage Publications.

[risa13165-bib-0020] Hofstede, G. (1991). Cultures and organizations: Software of the mind. London: McGraw‐Hill.

[risa13165-bib-0021] Hofstede, G. (2011). Dimensionalizing cultures: The Hofstede model in context. Online Readings in Psychology and Culture, 2(1). 10.9707/2307-0919.1014

[risa13165-bib-0022] Hofstede, G. , & Hofstede, G. J. (2005). Cultures and organizations: Software of the mind. New York: McGraw‐Hill.

[risa13165-bib-0023] Hofstede, G. , Hofstede, G. J. , & Minkov, M. (2010). Cultures and organizations: Software of the mind: Intercultural cooperation and its importance for survival. New York: McGraw‐Hill.

[risa13165-bib-0024] Hood, C. , Rothstein, H. , & Baldwin, R. (2001). The government of risk: Understanding risk regulation regimes. Oxford: Oxford University Press.

[risa13165-bib-0025] Hoppe, R. (2007). Applied cultural theory: Tool for policy analysis In Fischer F., Miller G. J., & Sidney M. S. (Eds.), Handbook of public policy analysis: Theory, politics, and methods. Boca Raton, FL: CRC Press.

[risa13165-bib-0026] Hoppe, R. (2009). Scientific advice and public policy: Expert advisers' and policymakers' discourses on boundary work. Poiesis & Praxis, 6(3–4), 235–263. 10.1007/s10202-008-0053-3 19655051 PMC2720174

[risa13165-bib-0027] IARC (International Agency for Research on Cancer) . (2017). Some organophosphate insecticides and herbicides. Lyon, France: International Agency for Research on Cancer Retrieved from https://monographs.iarc.fr/ENG/Monographs/vol112/mono112.pdf. 31829533

[risa13165-bib-0028] IPCS (International Programme on Chemical Safety) . (2004). IPCS risk assessment terminology. Geneva: World Health Organization Retrieved from https://www.who.int/ipcs/methods/harmonization/areas/ipcsterminologyparts1and2.pdf?ua=1.

[risa13165-bib-0029] IRGC (International Risk Governance Council) . (2005). Risk governance: Towards an integrative approach. Geneva: International Risk Governance Council.

[risa13165-bib-0030] IRGC (International Risk Governance Council) (2006). White paper on nanotechnology risk governance. Geneva: International Risk Governance Council.

[risa13165-bib-0031] IRGC (International Risk Governance Council) (2008). An introduction to the IRGC risk governance Framework. Geneva: International Risk Governance Council.

[risa13165-bib-0032] IRGC (International Risk Governance Council) (2009). Risk governance of synthetic biology—Concept note. Geneva: International Risk Governance Council.

[risa13165-bib-0033] IRGC (International Risk Governance Council) (2017). Introduction to the IRGC risk governance framework, revised version. Lausanne: EPFL International Risk Governance Center.

[risa13165-bib-0034] Jann, W. , & Wegrich, K. (2007). Theories of the policy cycle In Fischer F., Miller G. J., & Sidney M. S. (Eds.), Handbook of public policy analysis: Theory, politics, and methods. Boca Raton, FL: CRC Press.

[risa13165-bib-0035] Jasanoff, S. (1990). The fifth branch. Cambridge, MA: Harvard University Press.

[risa13165-bib-0036] Jenkins‐Smith, H. C. , Nohrstedt, D. , Weible, C. M. , & Sabatier, P. A. (2014). The advocacy coalition framework: Foundations, evolution and ongoing research In Sabatier P. A. & Weible C. M. (Eds.), Theories of the policy process. Boulder, CO: Westview Press.

[risa13165-bib-0037] Jenkins‐Smith, H. C. , Silva, C. L. , Gupta, K. , & Ripberger, J. T. (2014). Belief system continuity and change in policy advocacy coalitions: Using cultural theory to specify belief systems, coalitions, and sources of change. Policy Studies Journal, 42(4), 484–508. 10.1111/psj.12071

[risa13165-bib-0038] Johannesson, M. , Hansson, S. O. , Rudén, C. , & Wingborg, M. (1999). Risk management—The Swedish way(s). Journal of Environmental Management, 57(4), 267–281. 10.1006/jema.1999.0300

[risa13165-bib-0040] Kasperson, J. X. , Kasperson, R. E. , Pidgeon, N. , & Slovic, P. (2003). The social amplification of risk: Assessing fifteen years of research and theory In Pidgeon N., Kasperson R. E., & Slovic P. (Eds.), The social amplification of risk. Cambridge: Cambridge University Press.

[risa13165-bib-0039] Kasperson, R. E. , Renn, O. , Slovic, P. , Brown, H. S. , Emel, J. , Goble, R. , … Ratick, S. (1988). The social amplification of risk: A conceptual framework. Risk Analysis, 8(2), 177–187. 10.1111/j.1539-6924.1988.tb01168.x

[risa13165-bib-0041] Lebret, E. (2016). Integrated environmental health impact assessment for risk governance purposes; across what do we integrate? International Journal of Environmental Research and Public Health, 13(71), 1–15. 10.3390/ijerph13010071 PMC4730462 26703709

[risa13165-bib-0042] Löfstedt, R. E. (2011). Risk versus hazard—How to regulate in the 21st century. European Journal of Risk Regulation, 2(2), 149–168. 10.1017/S1867299X00001033

[risa13165-bib-0043] Löfstedt, R. E. , & Renn, O. (1997). The Brent Spar controversy: An example of risk communication gone wrong. Risk Analysis, 17(2), 131–136. 10.1111/j.1539-6924.1997.tb00852.x

[risa13165-bib-0044] NRC (National Research Council) (1983). Risk assessment in the federal government: Managing the process. Washington, DC: National Academy Press. 25032414

[risa13165-bib-0045] NRC (National Research Council) (1996). Understanding risk: Informing decisions in a democratic society. Washington, DC: National Academy Press.

[risa13165-bib-0046] NRC (National Research Council) (2009). Science and decisions: Advancing risk assessment. Washington, DC: National Academy Press. 25009905

[risa13165-bib-0047] Pielke, R. A. J. (2007). The honest broker: Making sense of science in policy and politics. Cambridge: Cambridge University Press.

[risa13165-bib-0048] Rayner, S. (1992). Cultural theory and risk analysis In Krimsky S. & Golding D. (Eds.), Social theories of risk. Westport, CT: Praeger.

[risa13165-bib-0049] Redmayne, M. (2016). International policy and advisory response regarding children's exposure to radio frequency electromagnetic fields (RF‐EMF). Electromagnetic Biology and Medicine, 35(2). 10.3109/15368378.2015.1038832 26091083

[risa13165-bib-0050] Renn, O. (2008). Risk governance: Coping with uncertainty in a complex world. London: Earthscan.

[risa13165-bib-0051] Rothstein, H. F. , Demeritt, D. , Paul, R. , Beaussier, A. ‐L. , Wesseling, M. , Howard, M. , … Bouder, F. (2017). Varieties of risk regulation in Europe: Coordination, complementarity and occupational safety in capitalist welfare states. Socio‐Economic Review, 1–28. 10.1093/ser/mwx029

[risa13165-bib-0052] Sabatier, P. A. (1998). The advocacy coalition framework: Revisions and relevance for Europe. Journal of European Public Policy, 5(1), 98–130. 10.1080/13501768880000051

[risa13165-bib-0053] Sabatier, P. A. , & Jenkins‐Smith, H. (1993). Policy change and learning: An advocacy coalition framework. Boulder, CO: Westview.

[risa13165-bib-0054] Sarewitz, D. (2004). How science makes environmental controversies worse. Environmental Science and Policy, 7, 385–403. 10.1016/j.envsci.2004.06.001

[risa13165-bib-0055] SCHER/SCENHIR/SCCS (Scientific Committee on Health and Environmental Risks/Scientific Committee on Emerging and Newly Identified Health Risks/Scientific Committee on Consumer Safety) (2013). Making risk assessment more relevant for risk management. Brussels: European Commission.

[risa13165-bib-0056] Schwartz, M. , & Thompson, M. (1990). Divided we stand: Redefining politics, technology and social choice. Philadelphia: University of Pennsylvania Press.

[risa13165-bib-0057] Siegrist, M. , Keller, C. , & Kiers, H. A. L. (2005). A new look at the psychometric paradigm of perception of hazards. Risk Analysis, 25(1), 211–222. 10.1111/j.0272-4332.2005.00580.x 15787770

[risa13165-bib-0058] Simon, H. A. (1957). Models of man: Mathematical essays on rational human behavior in a social setting. New York: John Wiley & Sons.

[risa13165-bib-0059] Slovic, P. (1987). Perception of risk. Science, 236, 280–285. 10.1126/science.3563507 3563507

[risa13165-bib-0060] Slovic, P. (1999). Trust, emotion, sex, politics, and science: surveying the risk‐assessment battlefield. Risk Analysis, 19(4), 689–701. 10.1111/j.1539-6924.1999.tb00439.x 10765431

[risa13165-bib-0061] Slovic, P. , Fischhoff, B. , & Lichtenstein, S. (1976). Cognitive processes and societal risk taking In Carroll J. S. & Payne J. W. (Eds.), Cognition and social behavior. London: Lawrence Erlbaum Associates, Inc.

[risa13165-bib-0062] Slovic, P. , Fischhoff, B. , & Lichtenstein, S. (1982). Why study risk perception? Risk Analysis, 2(2), 83–93. 10.1111/j.1539-6924.1982.tb01369.x

[risa13165-bib-0064] Spruijt, P. (2016). *Expert views on scientific policy advice on complex environmental health issues* . PhD dissertation, Utrecht University, Utrecht.

[risa13165-bib-0065] Spruijt, P. , Knol, A. B. , Petersen, A. C. , & Lebret, E. (2015). Different roles of electromagnetic field experts when giving policy advice: An expert consultation. Environmental Health, 14(7), 1–10. 10.1186/1476-069X-14-7 25604825 PMC4417251

[risa13165-bib-0066] Spruijt, P. , Knol, A. B. , Petersen, A. C. , & Lebret, E. (2016). Differences in views of experts about their role in particulate matter policy advice: Empirical evidence from an international expert consultation. Environmental Science and Policy, 59, 44–52. 10.1016/j.envsci.2016.02.003

[risa13165-bib-0067] Spruijt, P. , Knol, A. B. , Torenvlied, R. , & Lebret, E. (2013). Different roles and viewpoints of scientific experts in advising on environmental health risks. Risk Analysis, 33(10), 1844–1857. 10.1111/risa.12020 23465050

[risa13165-bib-0068] Spruijt, P. , Knol, A. B. , Vasileiadou, E. , Devilee, D. , Lebret, E. , & Petersen, A. C. (2014). Roles of scientists as policy advisers on complex issues: A literature review. Environmental Science & Policy, 40, 16–25. 10.1016/j.envsci.2014.03.002

[risa13165-bib-0063] Tansey, J. , & O'Riordan, T. (1999). Cultural theory and risk: A review. Health, Risk and Society, 1(1), 71–90. 10.1080/13698579908407008

[risa13165-bib-0069] Tversky, A. , & Kahneman, D. (1973). Availability: A heuristic for judging frequency and probability. Cognitive Psychology, 5, 207–232. 10.1016/0010-0285(73)90033-9

[risa13165-bib-0070] Tversky, A. , & Kahneman, D. (1974). Judgment under uncertainty: Heuristics and biases. Science, 185(4157), 1124–1131. 10.1126/science.185.4157.1124 17835457

[risa13165-bib-0071] van Asselt, M. B. A. , & Renn, O. (2011). Risk governance. Journal of Risk Research, 12(4), 431–449. 10.1080/13669877.2011.553730

[risa13165-bib-0072] Weible, C. M. , & Sabatier, P. A. (2007). A guide to the advocacy coalition framework In Fischer F., Miller G. J., & Sidney M. S. (Eds.), Handbook of public policy analysis: Theory, politics, and methods. Boca Raton, FL: CRC Press.

[risa13165-bib-0073] Weible, C. M. , Sabatier, P. A. , & McQueen, K. (2009). Themes and variations: Taking stock of the advocacy coalition framework. Policy Studies Journal, 37(1), 121–140. 10.1111/j.1541-0072.2008.00299.x

[risa13165-bib-0074] Weiss, C. (2003). Scientific uncertainty and science‐based precaution. International Environmental Agreements: Politics, Law and Economics, 3(2), 137–166. 10.1023/A:1024847807590

[risa13165-bib-0075] Wiener, J. B. , Rogers, M. D. , Hammitt, J. K. , & Sand, P. H. (2011). The reality of precaution: Comparing risk regulation in the United States and Europe. London: RFF Press.

[risa13165-bib-0076] WRR (The Netherlands Scientific Council for Government Policy) (2012). Physical safety: A matter of balancing responsibilities. The Hague: The Netherlands Scientific Council for Government Policy.

